# Molecular Characterization of *Cryptosporidium* Species and *Giardia duodenalis* from Symptomatic Cambodian Children

**DOI:** 10.1371/journal.pntd.0004822

**Published:** 2016-07-07

**Authors:** Catrin E. Moore, Kristin Elwin, Nget Phot, Chanthou Seng, Saroeun Mao, Kuong Suy, Varun Kumar, Johanna Nader, Rachel Bousfield, Sanuki Perera, J. Wendi Bailey, Nicholas J. Beeching, Nicholas P. J. Day, Christopher M. Parry, Rachel M. Chalmers

**Affiliations:** 1 Mahidol-Oxford Tropical Medicine Research Unit, Faculty of Tropical Medicine, Mahidol University, Bangkok, Thailand; 2 Cambodia-Oxford Medical Research Unit, Angkor Hospital for Children, Siem Reap, Cambodia; 3 Modernising Medical Microbiology Consortium, Nuffield Department of Clinical Medicine, John Radcliffe Hospital, University of Oxford, Oxford, United Kingdom; 4 Centre for Tropical Medicine and Global Health, Nuffield Department of Medicine, University of Oxford, Oxford, United Kingdom; 5 Cryptosporidium Reference Unit, Public Health Wales Microbiology, Singleton Hospital, Swansea, United Kingdom; 6 Swansea University Medical School, Grove Building, Swansea University, Singleton Park, Swansea, United Kingdom; 7 Angkor Hospital for Children, Siem Reap, Cambodia; 8 Clinical Sciences Group, Liverpool School of Tropical Medicine, Liverpool, United Kingdom; 9 Microbiology Department, Addenbrooke’s Hospital, Cambridge, United Kingdom; 10 NIHR Health Protection Research Unit (HPRU) in Gastrointestinal Infections, University of Liverpool, Liverpool, United Kingdom; 11 London School of Hygiene and Tropical Medicine, London, United Kingdom; 12 School of Tropical Medicine and Global Health, Nagasaki University, Nagasaki, Japan; QIMR Berghofer Medical Research Institute, AUSTRALIA

## Abstract

**Background:**

In a prospective study, 498 single faecal samples from children aged under 16 years attending an outpatient clinic in the Angkor Hospital for Children, northwest Cambodia, were examined for *Cryptosporidium* oocysts and *Giardia* cysts using microscopy and molecular assays.

**Methodology/Principal Findings:**

*Cryptosporidium* oocysts were detected in 2.2% (11/498) of samples using microscopy and in 7.7% (38/498) with molecular tests. *Giardia duodenalis* cysts were detected in 18.9% (94/498) by microscopy and 27.7% (138/498) by molecular tests; 82% of the positive samples (by either method) were from children aged 1–10 years. *Cryptosporidium hominis* was the most common species of *Cryptosporidium*, detected in 13 (34.2%) samples, followed by *Cryptosporidium meleagridis* in 9 (23.7%), *Cryptosporidium parvum* in 8 (21.1%), *Cryptosporidium canis* in 5 (13.2%), and *Cryptosporidium suis* and *Cryptosporidium ubiquitum* in one sample each. *Cryptosporidium hominis* and *C*. *parvum* positive samples were subtyped by sequencing the GP60 gene: *C*. *hominis* IaA16R6 and *C*. *parvum* IIeA7G1 were the most abundant subtypes. *Giardia duodenalis* was typed using a multiplex real-time PCR targeting assemblages A and B. Assemblage B (106; 76.8% of all *Giardia* positive samples) was most common followed by A (12.3%) and mixed infections (5.1%). Risk factors associated with Cryptosporidium were malnutrition (AOR 9.63, 95% CI 1.67–55.46), chronic medical diagnoses (AOR 4.51, 95% CI 1.79–11.34) and the presence of birds in the household (AOR 2.99, 95% CI 1.16–7.73); specifically *C*. *hominis* (p = 0.03) and *C*. *meleagridis* (p<0.001) were associated with the presence of birds. The use of soap was protective against *Giardia* infection (OR 0.74, 95% CI 0.58–0.95).

**Conclusions/Significance:**

This is the first report to describe the different *Cryptosporidium* species and subtypes and *Giardia duodenalis* assemblages in Cambodian children. The variety of *Cryptosporidium* species detected indicates both anthroponotic and zoonotic transmission in this population. Interventions to improve sanitation, increase hand washing after defecation and before preparing food and promote drinking boiled water may reduce the burden of these two parasites.

## Author Summary

The parasites *Cryptosporidium* and *Giardia* frequently cause diarrhea in children worldwide. They can be transmitted from animals to humans and between humans, either through direct contact, from sewage, or by contamination of food and water. The importance of these infections in Cambodian children is unknown. Animals are an essential part of life in Southeast Asia with livestock as well as domestic animals living in close proximity to humans. We previously conducted a prospective study to determine the intestinal parasites causing disease in symptomatic children attending hospital in Siem Reap, Northwestern Cambodia. This study examines these two parasites in more detail using molecular methods which provide better detection and are more discriminating. We were able to determine the different”strains” of *Cryptosporidium* and *Giardia* and, along with risk factor data, concluded that most of the infections were spread between people or within households suggesting that public health programmes should concentrate on improved sanitation, community handwashing initiatives and better food and water hygiene practices.

## Introduction

The protozoan genera *Cryptosporidium* and *Giardia* are important causes of diarrhoea worldwide [2–4]. Estimates of the prevalence of infection with these parasites vary enormously due to difficulties in diagnosis and reporting, and this is a particular problem in developing countries [5]. Cryptosporidiosis infection causes diarrhoea and vomiting, abdominal cramps, loss of appetite and low-grade fever. Giardiasis infection is associated with diarrhoea, stomach cramps, bloating, nausea, fatigue and weight loss during chronic infections [6]. Chronic infections with these pathogens contribute to malnutrition, poor growth and subsequent lack of achievement. Both diseases are more common in children than adults. *Cryptosporidium* and *Giardia* are parasites of humans, domestic animals and wild vertebrates, and the oocysts and cysts shed in the faeces are ubiquitous in the environment. Transmission occurs through anthroponotic, zoonotic, foodborne and waterborne routes [7–10]. The relative importance of transmission routes for each parasite is unclear due to the lack of sensitive tools to detect and then determine the species or subspecies during infection and in the environment.

There are more than twenty seven known *Cryptosporidium* species. The most common causing disease in humans are *Cryptosporidium parvum*, *C*. *hominis*, *C*. *meleagridis*, *C*. *felis*, *C*. *canis* and *C*. *ubiquitum* [11]. The species distribution varies geographically and temporally. In most countries *C*. *parvum* and/or *C*. *hominis* are the predominant human infective species. The only known *Giardia* species causing human disease is *Giardia duodenalis* (also known as *Giardia lamblia* and *Giardia intestinalis*). *Giardia duodenalis* is separated into assemblages identified originally by enzyme electrophoretic profiles but which are now determined by molecular analyses. There are seven assemblages (A-H), many of which are host adapted, A and B being the ones most frequently found in humans [12, 13].

Few data are available on the different *Cryptosporidium* species and *G*. *duodenalis* assemblages causing disease in children in Cambodia. Routine diagnostic facilities do not provide this level of differentiation. *Giardia duodenalis* can be diagnosed using direct microscopy of a wet preparation of faeces, with or without concentration. *Cryptosporidium* species are usually detected by direct microscopy of a cold Zeihl Neelson (ZN) faecal smear. These direct microscopy methods are widely used in both developed and developing countries but are insensitive. Antigen-detecting enzyme immunoassays (EIA) can also be used directly on faeces. Nucleic acid amplification methods, including real-time PCR assays, increase diagnostic sensitivity and also provide the opportunity for genotyping for epidemiological purpose and allow a better understanding of the routes of transmission [14].

In this study we determined the occurrence of *Cryptosporidium* spp. and *Giardia duodenalis* in the faeces of 498 symptomatic children attending the Angkor Hospital for Children, Siem Reap, North Western Cambodia. In addition we used PCR-based methods to genotype the parasites.

## Methods

### Study setting

This is a sub-study of a prospective investigation of faecal parasites in children attending Angkor Hospital for Children (AHC) in Siem Reap, North-Western Cambodia. AHC is a 50-bedded, charitably-funded paediatric hospital, providing free intensive, surgical and general medical care to children <16 years of age from Siem Reap town and surrounding provinces. The main study has been previously reported [1].

### Ethical consideration and treatment

The study was approved by the Institutional Review Board at AHC, the Oxford Tropical Research Ethics Committee (OXTREC 12–12) and the Public Health Wales Research Risk Review Committee.

### Ethics statement

The study was explained to patients and their caregivers, and informed consent was confirmed by the caregiver’s signature or a witnessed thumbprint. The attending physician recorded clinical and demographic information on a standard form together with potential risk factors for infection [1].

### Clinical, demographic and epidemiological data collection

Children attending the out-patient clinic or admitted to hospital between 3^rd^ April 2012 and 29^th^ June 2012 were eligible for inclusion in the study if the treating doctor had requested a faecal parasite examination because of diarrhoea, abdominal pain, clinical evidence of anaemia or malnutrition.

### Laboratory methods

Faecal samples were examined within one hour of receipt by direct microscopy of a wet preparation [15] and a faecal concentration (FC) preparation using the Evergreen faecal parasite concentrator (Evergreen Scientific, Los Angeles, California) [1]. *Giardia* cysts were detected by examination of both the direct and concentrated samples and *Cryptosporidium* oocysts using a ZN stained smear prepared from the concentrated deposit [16]. The faeces were then stored at -80°C and later shipped on dry ice to the University of Oxford.

A total of 498 of the original 865 patient samples were selected for this sub-study. The samples chosen included all samples known to be direct microscopy-positive for *Cryptosporidium* and *Giardia* and 395 samples which were direct microscopy negative. Three samples (one positive for *Cryptosporidium*, one positive for *Giardia* and one negative) were included in duplicate to test the reproducibility of all methods. DNA extraction was performed on the chosen samples in Oxford and the DNA was stored at -80°C until shipped on dry ice to Swansea where it was stored at -20°C prior to analysis.

### Molecular methods

#### DNA extraction

Frozen faecal samples were thawed at room temperature and DNA was extracted using the QIAamp Stool Mini Kit (Qiagen, UK) with modifications to increase the yield of DNA [17]. Briefly, 200μl of stool was added to 1ml of Buffer ASL, vortexed until the sample was homogenized. Samples underwent five cycles of immersion in liquid nitrogen for two minutes followed by heating at 95°C for two minutes, then samples were lysed at 95°C for ten minutes, cooled and vortexed for 15 seconds, followed by centrifugation at 13,000g for one minute. The supernatant was removed and the protocol was followed as per the manufacturer’s instructions.

#### Identification and characterization of *Cryptosporidium* species

To identify the *Cryptosporidium* species two assays were used. The samples were first screened using the 18S (SSU rRNA gene) real time PCR as described by Hadfield *et al*. [18]; positive samples then underwent a multiplex real-time PCR for the specific identification of *C*. *parvum* and *C*. *hominis* [18]. Primers used were specific for the Lib13 locus of *C*. *parvum*, as described previously [18], and the A135 locus of *C*. *hominis* (forward primer (CRUAF) 5’-CACCAAAGATAATGGATGTTGTTGAT-3’, reverse primer (CRUARb) 5’-AATTGCTTCGACATCGTCCAAT-3’ and probe (CRUAT) 6-FAM CAAACGAGCTATTAAAGG MGB-NFQ (personal communication, RC).

The PCR conditions were as described previously [18] and the post run analysis was performed using the Rotorgene 6000 software program, version 1.7 (Corbett Research, UK). Samples positive by the 18S assay but negative for *C*. *parvum* or *C*. *hominis* were sequenced. Samples positive for *C*. *parvum* and *C*. *hominis* by the multiplex assay were subtyped by sequencing part of the gp60 gene [19].

#### Identification of *Giardia duodenalis* assemblages

Two PCRs were performed. First a screening assay based on the beta-giardin gene (“GDD” assay) was performed on all samples using forward (BGF2) 5’-GAGGCCCTCAAGAGCCTGAA-3’, and reverse primers (BG2R2) 5’-ACACTCGACGAGCTTCGTGTT-3’ with probe (BGT2) VIC-ATCGAGAAGGAGACGATCGC-MGB-NFQ (personal communication, RC) using conditions described previously [17]. Samples positive in the GDD screening assay underwent a multiplex real time PCR based on the *tpi* locus, specific for assemblages A and B [17] and sequencing of the GDD PCR products.

### Sequencing

The PCR products for *Cryptosporidium* species and *Giardia* species were purified using 1.8μl Agencort Ampure XP beads (Becman Coulter, UK) per 1μl of PCR product. Samples were mixed, incubated and transferred to a magnetic holder, the supernatant removed, the beads washed twice with 200μl of 80% ethanol. The ethanol was then removed and the DNA/beads dried. The beads were eluted in 26μl Tris-EDTA buffer, resuspended, returned to the magnetic holder and 25μl of cleaned DNA was removed.

The sequencing reaction contained 1–3ng of DNA, 1.75μl sequence dilution buffer, 0.5μl BigDye, 0.5μl of forward or reverse primer (18S for *Cryptosporidium* species and GDD for *Giardia* species) with water up to 10μl. The cycling conditions were: initial denaturation of 96°C for 2 minutes followed by 30 cycles consisting: 96°C for 10 seconds, 50°C for 5 seconds and 60°C for 3 minutes.

The products for sequencing were purified using 10μl of Agencourt CleanSEQ (Beckman Coulter, UK) beads per reaction, washed with 85% ethanol, placed on a magnetic strip and the supernatant was discarded. The beads were then washed twice with 85% ethanol, the samples were air dried and the resulting product was eluted in 0.1M EDTA. The sequencing reactions were performed on an ABI3730 automated sequencer (Applied Biosystems, ThermoFisher, UK). Consensus sequences for each strain were prepared using the Genious 8.0.3 software (Biomatters Ltd.) and compared with the published sequences available in GenBank using BLAST.

### Statistical analysis

The sensitivity and specificity of the detection by microscopy of each parasite was calculated using the PCR result as the nominated gold standard. Study participants were subdivided into five age groups for analysis: neonates (≤28 days); infants (29 days-<1 year); 1–5 years; 6–10 years and 11–16 years. A chi-squared test or Fisher’s Exact test was used to examine the association between categorical variables. The association of potential risk factors with *Cryptosporidium* species and *Giardia duodenalis* detection was analysed using univariate and multivariate logistic regression methods using an odds ratio with 95% confidence intervals. Statistical analyses were performed using STATA version 13.1 (Stata Corporation; College Station, TX, USA).

## Results

### Detection of *Cryptosporidium* and *Giardia* species

When the 498 samples were examined by microscopy *Cryptosporidium* oocysts were detected in 11/498 (2.2%) and *Giardia* cysts in 94/498 (18.9%). The additional use of the molecular methods significantly increased the total number of samples identified as positive for *Cryptosporidium spp* to 38/498 (7.7%) (p<0.001) and for *Giardia duodenalis* to 143/498 (28.7%) (p<0.001) (Table 1). There was agreement between the results of microscopy and molecular detection in 471/498 (94.6%) samples for *Cryptosporidium* spp and 438/498 (88.0%) for *G*. *duodenalis*. Of the 38 samples positive by either method for *Cryptosporidium* species, none were positive by microscopy alone, 11 (28.9%) were positive by both methods and 27 (71.1%) were positive by PCR alone. For 143 samples positive by either method for *Giardia*, 11 (7.7%) were positive by microscopy alone, 49 (34.3%) were positive by PCR alone, and 83 (58.0%) were positive by both microscopy and PCR (Table 1). All duplicate samples were exactly the same on both occasions.

**Table 1:** Detection of *Cryptosporidium* spp. and *Giardia duodenalis*.

	*Cryptosporidium* species				*G*. *duodenalis*
				Sensitivity (95% CI)	Specificity (95% CI)
		*PCR positive (%)*	*PCR negative (%)*		
***Cryptosporidium* spp.**	*Microscopy positive (ZN*^*+*^*)*	11 (2.2)	0	100 (71.5–100)	94.5 (92.0–96.3)
	*Microscopy negative (ZN*^*-*^*)*	27 (5.5)	460 (94.5)		
***Giardia duodenalis***	*Microscopy positive*	83 (16.7)	11 (2.2)	88.3 (80.0–94.0)	87.9 (84.3–90.9)
	*Microscopy negative*	49 (9.8)	355 (71.3)		

### *Cryptosporidium* species identification

Six *Cryptosporidium* species were detected (Table 2). Of the 38 *Cryptosporidium* positive samples, 13 (34%) were *C*. *hominis* with the most prevalent being *C*. *hominis* subtype IaA16R6 (23.1% of all *C*. *hominis*); 9 (23.7%) were *C*. *meleagridis*; 8 (21.1%) were *C*. *parvum*, with subtype IIeA7G1 found in 50% of the *C*. *parvum* samples; 5 (13.2%) were *C*. *canis*; and single positives found for *C*. *suis* and *C*. *ubiquitum*. One sample was positive with a mixture of *C*. *hominis* and *C*. *parvum* so the subtypes failed to be determined.

**Table 2:** Univariate and multivariate risk factor analyses for infections caused *Cryptosporidium* spp. and *Giardia duodenalis*, data available in S2 Table.

Variable	Univariate analysis			Multivariate analysis			Univariate analysis		
	Odds ratio [OR]	95% CI	p-value	Odds ratio [OR]	95% CI	p-value	OR	95% CI	p-value
Male gender	1.15	0.59–2.23	0.68				1.01	0.68–1.50	0.96
Age group^1^	0.74	0.52–1.06	0.10				1.11	0.90–1.39	0.33
*Location of residence*									
In Siem Reap town	1.13	0.52–2.47	0.76				0.74	0.45–1.24	0.26
Outside Siem Reap town	0.79	0.37–1.68	0.54				1.32	0.80–2.16	0.28
*Risk factors*									
*Clinical syndromes*									
Diarrhoea	1.15	0.58–2.29	0.69				1.01	0.67–1.54	0.95
Abdominal pain duration	1.00	0.47–2.14	1.00				1.51	0.94–2.14	0.09
Anaemia Hb (g/dL) if present	1.05	0.39–2.80	0.92				0.69	0.36–1.32	0.26
Malnutrition	**4.84**	**2.00–11.70**	**<0.0001**	**9.63**	**1.67–55.46**	**0.01**	0.62	0.25–1.55	0.31
Presence of wasting	**4.13**	**1.55–10.99**	**0.005**	0.28	0.04–2.11	0.22	0.49	0.17–1.45	0.20
Chronic medical diagnoses	**5.4**	**2.31–12.63**	**<0.0001**	**4.51**	**1.79–11.34**	**0.001**	**0.25**	**0.08–0.84**	**0.02**
*Domestic animals at home*	0.76	0.37–1.56	0.46				0.90	0.58–1.41	0.64
Cat	0.88	0.44–1.74	0.70				1.02	0.68–1.53	0.91
Dog	0.51	0.26–0.99	0.05				0.93	0.62–1.40	0.72
Birds	**3.02**	**1.23–7.39**	**0.016**	**2.99**	**1.16–7.73**	**0.02**	0.82	0.38–1.78	0.61
*Livestock at home*	0.67	0.34–1.30	0.24				0.91	0.60–1.39	0.67
Water buffalo	-	-	-				1.11	0.34–3.61	0.86
Chickens	0.88	0.45–1.72	0.72				0.86	0.58–1.28	0.46
Pigs	0.44	0.13–1.47	0.18				0.95	0.55–1.65	0.85
Cattle	0.49	0.20–1.20	0.12				1.10	0.70–1.71	0.69
Ducks	6.19	0.55–69.86	0.14				-	-	-
*Main source of drinking water*									
River	1.43	0.48–4.26	0.52				0.95	0.45–2.01	0.90
Rain	-	-	-				1.08	0.44–2.65	0.86
Well	0.65	0.31–1.39	0.27				1.39	0.82–2.35	0.23
bottled	1.71	0.49–5.98	0.40				0.68	0.25–1.85	0.45
City	0.34	0.05–2.54	0.29				0.55	0.22–1.36	0.20
Pond	1.89	0.62–5.71	0.26				0.96	0.42–2.21	0.93
Water available at house	1.21	0.49–2.98	0.68				0.64	0.39–1.04	0.07
Ever wash hands with soap after defecation	1.02	0.67–1.55	0.93				0.74	0.58–0.95	**0.02**
Do not wash hands with soap after defecation	0.92	0.39–2.16	0.85				1.97	1.23–3.15	**0.005**
*Place of defaecation*									
Toilet	1.28	0.66–2.51	0.47				0.78	0.53–1.17	0.23
Forest	0.69	0.33–1.46	0.33				1.13	0.75–1.72	0.56
Farm	0.37	0.13–1.07	0.07				0.72	0.44–1.18	0.19
Outside house	0.74	0.34–1.60	0.45				0.93	0.60–1.45	0.75
In the river	1.43	0.48–4.26	0.52				0.95	0.45–2.01	0.90
Wears shoes	0.51	0.24–1.08	0.08				0.77	0.46–1.28	0.31
Goes to school	0.73	0.09–6.07	0.77				0.93	0.38–2.78	0.93

^1^Study participants were subdivided into five age groups for analysis: neonates (≤28 days); infants (29 days-<1 year); 1–5 years; 6–10 years and 11–16 years, none of these ages were significant in the analyses

Significant values are shown in bold (p<0.05)

### Giardia duodenalis sequencing

Only two *Giardia duodenalis* assemblages were found in 132 samples tested despite all possible assemblages being sought (Table 3). The most common was assemblage B, found in 106 (80.3%) samples followed by assemblage A, found in 17 (12.9%). A total of 9 (6.8%) samples were positive for both assemblages A and B. Seventeen samples amplified at the beta giardin gene, but were not positive with the *tpi* typing PCR, so sequencing of the beta giardin gene was used to identify the assemblage; all these amplicons were assemblage B.

**Table 3:** *Cryptosporidium* and *Giardia* PCR and sequencing results. This excludes 11 samples that were positive for *G*. *duodenalis* by microscopy, but negative by PCR.

PCR result	Number positive by microscopy; number positive by PCR (% of PCR positive samples)	GP60 subtype (number of samples)
*Cryptosporidium* species sequencing results		
*Cryptosporidium spp*.	11; 38	
*C*. *hominis*	5; 13 (34.2%)	IaA14R3 (1)
		IaA16R6 (3)
		IdA17 (1)
		IfA19G1 (1)
		Negative (6)
		Seq poor (1)
*C*. *meleagridis*	3; 9 (23.7)	Not done
*C*. *parvum*	3; 8 (21.1)	IIeA7G1 (4)
		Negative (4)
*C*. *canis*	0; 5 (13.2)	Not done
*C*. *suis*	0; 1 (2.6)	Not done
*C*. *ubiquitum*	0; 1 (2.6)	Not done
*C*. *hominis* and *C*. *parvum* mixture	0; 1 (2.6)	Not done
*G*. *duodenalis* sequencing results		
*G*. *duodenalis*	47; 132	
*G*. *duodenalis* assemblage B	43; 63 (80.3)	Assemblage B (n = 17)
*G*. *duodenalis* assemblage A	3; 14 (12.9)	Not done
*G*. *duodenalis* assemblage A and B	1; 6 (5.3)	Not done
*G*. *duodenalis*	0; 2 (1.5)	Not done

### Clinical, demographic and epidemiological data

The risk factors for the presence of *Cryptosporidium* and *Giardia* species in the faeces of individual children were examined (Table 2, all data available in S2 Table). In the univariate analysis the risk factors for infection with *Cryptosporidium* were malnutrition (OR 4.84, 95% CI 2.00–11.70), wasting (OR 4.13, 95% CI 1.55–10.99), chronic medical diagnoses (OR 5.40, 95% CI 2.31–12.63) and the presence of birds in the house (OR 3.02, 95% CI 1.23–7.39). Malnutrition (AOR 9.63, 95% CI 1.67–55.46), chronic medical diagnoses (AOR 4.51, 95% CI 1.79–11.34) and the presence of birds (AOR 2.99, 95% CI 1.16–7.73) remained associated in the multivariate analysis. The associations with presence of birds and in particular chickens were examined further and significant associations were found with *C*. *hominis* and *C*. *meleagridis* (p = 0.03 and p<0.001 respectively) with birds but no significant association was found between the different *Cryptosporidium* species and the presence of chickens at the residence (S1 Table). For *Giardia* the use of soap was protective (OR 0.74, 95% CI 0.58–0.95).

## Discussion

This is the first detailed study of *Cryptosporidium* species and subtype and *Giardia duodenalis* assemblage distribution in symptomatic Cambodian children. The addition of molecular methods to standard microscopy increased the proportion of *Cryptosporidium* species detected from 1.4% to 7.7% of all faecal samples, with three quarters of positive samples only detected by PCR. Most of the *Cryptosporidium* species positives were detected in the 1–10 year age group with two thirds in the 1–5 years age group and one quarter in the 6–10 year age group. The proportion of samples positive for *G*. *duodenalis* increased from 11.2% to 28.7% using molecular methods in addition to microscopy and one third were positive by PCR alone. In contrast to the *Cryptosporidium* species, most of the *Giardia* infections were detected in children aged 1–10 years, with 45% in the 1–5 year age group and 42% in the 6–10 year age group. Similar increased detection rates were described recently using multiplex PCR methods targeting *Cryptosporidium* species and *G*. *duodenalis*, although the prevalence was lower in the multiplex reaction than described here [20]. Other detailed reports from Cambodia have not examined faecal samples using PCR based techniques, therefore their prevalence detection rates are lower than observed in our study [21–23].

Six different *Cryptosporidium* species were found in this study in Cambodian children. The most common was *C*. *hominis*, an anthroponotic species known to be prevalent in developing countries [24]. The most common gp60 *C*. *hominis* subtype families previously reported in children in developing countries are Ia, Ib, Id and Ie [24–26]. In other studies in Malawi, Kenya, India and Australia IbA9G3 was common and IbA10G2 was common in South Africa, Peru, the UK and USA [27]. In contrast, subtypes IaA14R3, IaA16R6, IdA17 and IfA19G1 were detected in this study. There are few other reports describing these subtypes, although two reports have linked IaA14R3 subtype to outbreaks in Scotland [28] and elsewhere in the UK [29]. IaA16R6 was the most common subtype in this study but has not previously been described. IdA17 has been described in Nigeria, Scotland and Mexico [26, 28, 30] and IfA19G1 has been detected in small numbers in Australia [31].

The other *Cryptosporidium* species detected in these Cambodian samples are zoonotic: *C*. *parvum* suggests an animal reservoir (most often ruminant livestock); *C*. *meleagridis* is associated with different avian species [32]; *C*. *canis* infects dogs; *C*. *suis* infects pigs; and *C*. *ubiquitum* has a broad host range [33]. No associations were observed between the *Cryptosporidium* species and the animal reservoir in this study but the case numbers were small. *Cryptosporidium parvum* subtype IIa is the predominant subtype family in animals and humans worldwide and the IId subtype is reported frequently in Europe, Asia, Egypt and Australia [34]. The most widely distributed *C*. *parvum* subtype in industrialised countries is IIa15G2R1, with IIaA18G3R1 more common in Ireland [27]. All four of the *C*. *parvum* samples identified in this study were subtype IIeA7G1 which is rare; only a single strain has previously been described, from India [35]. Subtypes IIe and IIc are anthroponotic subtype families. IIeA7G1 was the most common subtype found in this study suggesting that anthroponotic transmission of *C*. *parvum* is important in Cambodian children. We also found nine patients with *C*. *meleagridis*, five patients with *C*. *canis* and one patient each with *C*. *suis* and *C*. *ubiquitum*. These species are also found in animals so while anthroponotic transmission of Cryptosporidium appears to be most important in children in Cambodia, there may also be a zoonotic reservoir of infection.

Only two *G*. *duodenalis* assemblages were present in these samples (Table 3). The most prevalent was assemblage B at 80%, with 13% assemblage A and 7% mixed A and B. The prevalence of assemblage B is consistent with previous data from India [36]. *Giardia duodenalis* assemblage B has previously been described as common in children [37]. Assemblages A and B are usually anthroponotically transmitted rather than from a zoonotic source, although dogs, cats and livestock have been associated with assemblage A [38]. The PCR testing strategy used in our study emphasizes the need to test samples with a number of typing methods. Although some samples that were positive using the beta giardin screening PCR, the *tpi* PCR method was unable to assign their assemblages and sequencing the beta giardin amplicons was used to determine the assemblages of 17 parasites, which were all assemblage B. Previous work showed the *tpi* assay to be sufficiently sensitive and specific so the reason is not known [19].

The study highlights the association of malnutrition, chronic medical diseases and the presence of domestic birds with *Cryptosporidium* infection. *Giardia duodenalis* infections were associated with not using soap, as described by others[39].

There are a number of study limitations. This analysis only used a subgroup of the larger prospective study [1] and so may not fully represent the disease burden and risk factors for *Cryptosporidium* and *Giardia* in the symptomatic children attending AHC. A small number of samples were positive for *Giardia* by microscopy but negative by PCR, as reported by other investigators [20, 40]. As both the DNA extraction and microscopy used a only a small sample of feces it is likely that by chance in some instances the parasite will be seen by microscopy but missed by PCR. The study included patients with symptoms but no asymptomatic controls. Some of the parasites may not be causing the child’s symptoms. We did not examine animals and so could not determine the distribution of zoonotic infections.

This study is the first to describe the anthroponotic and heterogeneous nature of *Cryptosporidium* species infecting children from North Western Cambodia. *Cryptosporidium hominis* was the most common species with four different subtypes, followed by *C*. *meleagridis* and *C*. *parvum* with a single subtype. The main *G*. *duodenalis* subtype was assemblage B. Further work is needed to fully describe the epidemiology of disease and the circulating subtypes of *Cryptosporidium* and *G*. *duodenalis* in Cambodia. This work reinforces the need for handwashing after defecation and the use of boiled water for consumption.

## Supporting Information

S1 Table Associations between the *Cryptosporidium* species and the presence of domestic birds or chicken at the residence. (DOCX)

S2 Table Raw data included in the risk factor analysis for *Cryptosporidium* spp. and *Giardia duodenalis*. Key: presence = 1, absence = 0.
(XLSX)
