# Supplementary material for: Molecular Characterization of Cryptosporidium Species and Giardia duodenalis from Symptomatic Cambodian Children
Source: PLoS Negl Trop Dis. 2016 Jul 7;10(7):e0004822. doi: 10.1371/journal.pntd.0004822 (PMC4936737; doi:10.1371/journal.pntd.0004822)
Supplement: S1 Table — (DOCX) [file pntd.0004822.s001.docx]

**S1 Table** Associations between the *Cryptosporidium* species and the presence of domestic birds or chicken at the residence

| *Cryptosporidium* species | Number | Presence of domestic birds at residence, total number (%) | P value | Presence of chickens at residence | P value |
| --- | --- | --- | --- | --- | --- |
| Negative | 458 | 31 (6.8) |  | 267 (58.3) |  |
| *C. canis* | 5 | 0 | 0.54 | 3 (60.0) | 0.94 |
| *C. hominis* | 13 | 3 (23.1) | **0.03** | 6 (46.2) | 0.38 |
| *C. hominis* and *C. parvum* | 1 | 0 | 0.79 | 1 (100.0) | 0.40 |
| *C. meleagridis* | 9 | 0 | 0.42 | 8 (88.9) | 0.07 |
| *C. parvum* | 8 | 4 (50.0) | **<0.001** | 3 (37.5) | 0.24 |
| *C. suis* | 1 | 0 | 0.79 | 0 | 0.24 |
| *C. ubiquitum* | 1 | 0 | 0.79 | 0 | 0.24 |
